# Role of Calixarene in Chemotherapy Delivery Strategies

**DOI:** 10.3390/molecules26133963

**Published:** 2021-06-29

**Authors:** Rossella Basilotta, Deborah Mannino, Alessia Filippone, Giovanna Casili, Angela Prestifilippo, Lorenzo Colarossi, Gabriele Raciti, Emanuela Esposito, Michela Campolo

**Affiliations:** 1 Department of Chemical, Biological, Pharmaceutical and Environmental Sciences, University of Messina, Viale Ferdinando Stagno D’Alcontres, 98166 Messina, Italy; robasilotta@unime.it (R.B.); debmannino@unime.it (D.M.); alessia.filippone@unime.it (A.F.); gcasili@unime.it (G.C.); campolom@unime.it (M.C.); 2 Istituto Oncologico del Mediterraneo, Via Penninazzo 7, 95029 Viagrande, Italy; angela.prestifilippo@grupposamed.com (A.P.); lorenzo.colarossi@grupposamed.com (L.C.); 3 IOM Ricerca Srl, Via Penninazzo 11, 95029 Viagrande, Italy; gabriele.raciti@grupposamed.com

**Keywords:** cancer, drug delivery system (DDS), calixarene, chemotherapeutic agents

## Abstract

Since cancer is a multifactorial disease with a high mortality rate, the study of new therapeutic strategies is one of the main objectives in modern research. Numerous chemotherapeutic agents, although widely used, have the disadvantage of being not very soluble in water or selective towards cancerous cells, with consequent side effects. Therefore, in recent years, a greater interest has emerged in innovative drug delivery systems (DDSs) such as calixarene, a third-generation supramolecular compound. Calixarene and its water-soluble derivatives show good biocompatibility and have low cytotoxicity. Thanks to their chemical–physical characteristics, calixarenes can be easily functionalized, and by itself can encapsulate host molecules forming nanostructures capable of releasing drugs in a controlled way. The encapsulation of anticancer drugs in a calixarene derivate improves their bioavailability and efficacy. Thus, the use of calixarenes as carriers of anticancer drugs could reduce their side effects and increase their affinity towards the target. This review summarizes the numerous research advances regarding the development of calixarene nanoparticles capable of encapsulating various anticancer drugs.

## 1. Introduction

Cancer is the second leading cause of death worldwide, after heart disease. According to a statistical study, in 2018 there were 3.9 million cases of cancer and 1.9 million deaths in Europe [1]. The cancers with the highest incidence are cancers of the female breast, followed by colorectal, lung and prostate cancer [2]. Despite the ongoing search for new strategies for cancer treatment, overall cancer-related mortality rates remain relatively stable. Currently, cancer prevalence is expected to increase in the near future, reaching 13.1 million cases in 2030 [3]. Pathological features of cancers, including no cell death and uncontrolled cell multiplication, have the ability to infiltrate into organs and tissues of the body by altering their structures and functionalities [4]. Traditional therapies, such as surgery, chemotherapy, radiotherapy and immunotherapy, have several side effects that can lead to systemic adverse effects [5]. In particular, many chemotherapy drugs are characterized by a low selectivity towards cancer cells and poor pharmacokinetic profiles. Furthermore, some anticancer drugs can lead to resistance phenomena or have a low half-life due to rapid degradation by enzymes. Because of this worrying situation, current efforts are focusing on the development of innovative technologies for the treatment of cancer, like the use of DDSs. These nanotechnologies predominantly use nanoscale materials and devices as vehicles that can easily cross biological barriers [6]. Over the past few years, several generations of drug delivery systems (DDSs) have been evaluated; today, we know three main kinds of nanocarriers: vesicular systems (like liposomes), particle systems (like polymers) and molecular systems (such as cyclodextrins and calixarene).

Among the DDSs used for the delivery of anticancer drugs, one of the most interesting molecules is calixarene. Calixarene allows the controlled and localized administration of anticancer drugs, increasing the effectiveness of delivery and consequently decreasing the side effects.

Furthermore, due to its extraordinary selectivity, calixarene targets cancer cells, avoids healthy cells damage and reduces side toxicity. For this reason, the use of anticancer chemotherapies within these carriers improves their biodistribution and tolerability [7]. The purpose of this review is to evaluate the role of calixarene, a macrocyclic third-generation molecule with extraordinary structural characteristics, in the delivery of anticancer drugs and delve into the main advantages of DDSs in anti-cancer therapies.

## 2. Drug Delivery Systems (DDSs)

DDSs are innovative technologies used for the delivery of active principles, characterized by site-specific targeting and delayed or prolonged release [8]. The DDSs are systems that can allow delayed and localized release at the cellular or tissue level. These systems are able to deliver a small amount of drug selectively to the site of action, protecting it from enzymatic degradation in biological fluids. Thus, on one hand, side effects are reduced due to the smaller amount of drug available, while on the other hand, the action of the drug is enhanced [9]. DDSs are classified into three generations [10]: the first generation was used to obtain zero-order release kinetics, keeping the concentration of the drug constant in the blood. Later, that idea was abandoned because the zero-order release was not correlated with the maintenance of a constant drug concentration in the blood. The first generation of these technologies includes numerous systems providing administration of short-acting drugs usually once or twice a day; the same mechanisms have also been used to develop transdermal patches once a day and once a week. Alternatively, the second generation of DDSs focused on the development of “intelligent” polymers and hydrogels to create delivery systems activated by changes in environmental factors, such as pH or temperature [11]. Finally, the third generation of DDSs provides an easier way of creating various amphiphilic compounds, which react to external stimuli thanks to the presence of weak and reversible non-covalent interactions. In particular, they are able to form host–guest interactions, facilitating the inclusion of drugs [12]. DDSs can also be classified into vesicular systems, particle systems and molecular systems. Vesicular systems contain liposomes, nanosomes, and virosomes. These have been widely applied in the development of new DDSs due to their unique structures and exceptional abilities to encapsulate and control drug delivery in different conditions. Liposomes are lipid-based nanovesicles characterized by having one or more double layers spontaneously obtained from the dispersion of natural or synthetic amphipathic lipids in water. They can encapsulate hydrophilic molecules in their internal aqueous phase, lipophilic drugs in the lipid membrane and amphiphilic drugs at the aqueous-lipid interface. Since the discovery of liposomes by Prof. Alec Bangham (1921–2010), numerous studies have been aimed at their clinical application [13]. Currently, more than 18 liposomal drugs have been approved for the clinical treatment of cancer and many other conditions. A representative liposomal drug is Doxil (liposomal doxorubicin), which consists of a doxorubicin (Dox) loaded onto a liposome modified with poly-ethylene-glycol (PEG). This nanovesicle allows a specific accumulation in tumor sites via enhanced permeation and retention (EPR) effects and reduces Dox-induced adverse events [14]. The second category of DDSs are nanoparticle systems that include, for example, polymeric nanoparticles (NPs), which can be produced through chemical processes such as nanoprecipitation or the double emulsion method. These chemical processes consist of the self-assembly of biodegradable copolymers with variable hydrophobicity which are appropriate for systemic administration. Thanks to their core-shell structure, polymeric NPs can easily encapsulate hydrophobic drugs and permit prolonged drug release into the systemic circulation. Genexol-PM is an example of a NPs formulation based on polymers of paclitaxel (PCX) and poly (*D*,*L*-lactide)-b-polyethylene glycol-methoxy (PLGA-mPEG), approved for the treatment of metastatic breast cancer [15]. DDSs of the third generation are represented by supramolecular systems such as cyclodextrins and calixarenes. First, cyclodextrins (CDs) are cyclic oligosaccharides composed by glucopyranose units and have internal hydrophobic cavities and external hydrophilic surfaces. CDs can be distinguished as α, β and γ derivatives based on the number of glucopyranose units (6, 7 or 8 units) [16]. One of the most used derivatives is certainly hydroxypropyl-β-cyclodextrin (HP-β-CD). This compound has an adequate depth of cavity and an appropriate size of the internal surface for the binding of drugs. For this reason, cyclodextrins are able to form host–guest inclusion complexes with many drugs, increasing their solubility in water. Consequently, these CDs have been shown to interact with cell membranes, reducing the activity of drug efflux pumps and conferring a powerful action against multidrug-resistant tumor cells [17]. In recent years, many studies have been performed to combine the benefits of cyclodextrins and liposomes by developing drug/cyclodextrin/liposome (DCL) inclusion complexes [18,19,20,21]. DCLs have been shown to improve the solubility of poorly water-soluble drugs such as paclitaxel, increasing their therapeutic efficacy in the treatment of multidrug-resistant tumors [22]. An in-depth study also developed a macro-complex consisting of calixarene and cyclodextrin. This compound has been synthesized to combine the advantages of both nanocarriers, thus obtaining a synergistic effect [23]. 

Particularly, these cyclodextrin-calixarene amphiphilic complexes are capable of self-assembling in nanospheres or nanovesicles and encapsulating hydrophobic anticancer agents such as docetaxel and temozolomide. It has been studied that the disassembly of the two molecules and the release of the drugs is mediated by high levels of glutathione, which is more expressed in the tumor microenvironment and thus leads to an increased selectivity of the anticancer drugs [24]. In this review we will particularly evaluate the role of calixarenes as compounds of great interest and importance for the development of intelligent drug delivery systems.

## 3. Calixarene: Structure and Applications

**Figure 1:**
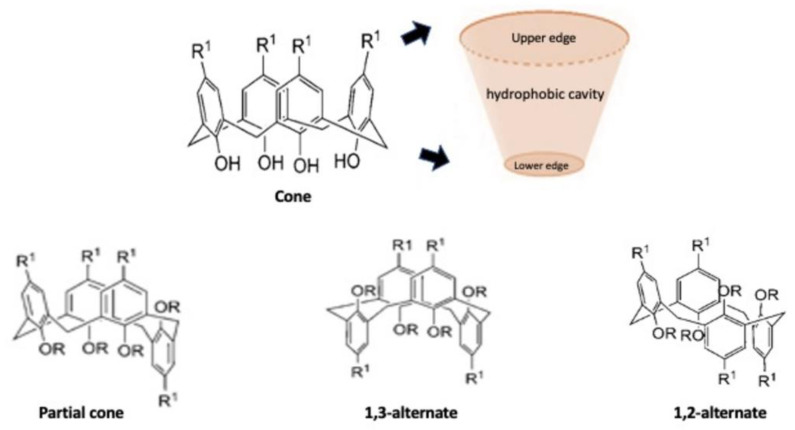
Schematic representation of functionalized structures of calixarene.

Calixarene is a macrocyclic molecule, that together with cyclodextrins, nanotubes, nanoparticles, micelles and corona ethers, represents the third generation of supramolecular compounds. Supramolecular chemistry is a discipline that studies self-assembly and reversible interactions of host–guest complexes for the construction of intelligent vehicles for drug delivery. These are intermolecular bonds, where species are linked together by non-covalent supramolecular interactions, including van der Waals forces, hydrophobic bonds, hydrogen bonding, metal-coordination and host–guest interactions. These interactions improve the stability of nanocarriers in biological fluids and optimize circulation time and transport performance. Despite having weak interactions, they are able to optimally bind the guests, making the structure more flexible and protecting the complex from small perturbations while maintaining the reversibility of the self-assembled structure [25]. These compounds can include appropriately selected drugs within them and can also be structurally modified to increase their affinity for a particular host [26]. This class of molecules was extensively studied for drug delivery because they present a variable conformation that makes them excellent drug carriers (Figure 1) [27]. 

In particular, calixarene is an organic compound synthesized by the reaction of para-substituted phenols with formaldehyde in basic or acidic conditions. In fact, the calixarenes, due to the presence of different phenolic units within their structure, can be considered cyclic polyphenols [28]. These are secondary metabolites, extensively studied for their beneficial effects on human health, especially as antioxidants, antiallergic, anti-inflammatory, antitumor, antihypertensive and antimicrobial agents and their uses in biomaterial engineering [29]. The compound is called calix [n] arene, where n is the number of phenolic units present in the macrocyclic unit [30]. Generally, the starting material used for the synthesis of calixarene is *p*-*tert*-butyl-calyx [n] arene, which can undergo chemical modifications in both lower and upper edges. The upper edge has tert-butyl groups which can be easily dealkylated, as to bind different functional groups as needed. Furthermore, the lower edge has four phenolic groups, –OH, which can be replaced with an alkyl chain, thus obtaining compounds with different properties [31]. This structure forms a hydrophobic cavity able to build host–guest inclusion complexes. The selectivity for the target can be increased through the functionalization of the calixarene. Compared with corona ethers and cyclodextrin, the functionalization of calixarene is easier to control and therefore represents an additional advantage. Calixarenes can also bind metal, metal ions, non-metallic oxides and organic molecules, including amino acids, amides, amines, alcohols, esters, aldehydes and alkyl derivatives, forming aggregates with different morphologies [32]. Moreover, some calixarene derivatives have shown important antibacterial, antifungal, antimicrobial and antitumor activities [33,34]. In fact, calixarene, besides the inclusion of molecules inside the cavity, can also create ditopic ligands with binding sites at the upper and lower edges, interacting with proteins and nucleic acids and modulating the activity of many enzymes, the proliferation of cancer cells and metabolic pathways [35]. A study shows that the calixarene molecule can be functionalized with *L*-proline leading to cell death by apoptosis, and shows a high toxicity against human colon cancer and lung cancer cells [36]. Hence, calixarenes possess other properties such as antimicrobial activity due to calix [4] arenes binding to the beta-lactam ring of penicillin as lower border substituents; in particular, penicillin V has been added via ether-ether junction to the *p*-*tert*-butylcalix [4] arene lower rim, showing efficacy against gram-positive and gram-negative bacteria [37].

Today, the versatility of the chemical and structural aspects of calixarene has prompted researchers to explore new fields of application. These macrocyclic nano-capsules linked to metals have shown relevant action in biomedical applications, and in particular in imaging and drug administration [29]. Furthermore, it has been demonstrated that a nanohydrogel constructed from a calix [4] arene macrocycle can be used as a curcumin release vehicle. The complex was topically administered in mice showing a powerful effect counteracting inflammatory process induced by imiquimod application in an in vivo model of psoriasis, without toxicity [38]. Moreover, the synthesis of novel nonionic amphiphilic dendro-calix [4] arene compounds, which increased the solubility of hydrophobic drugs such as naproxen and ibuprofen, has been reported. Thus, it has been shown that the length of the alkyl chain and the intrinsic cyclic nature of the calixarene structure particularly affect the solubility of the drugs tested [39]. It is important to consider also that the solubility of the macrocycle, according to Lipinski’s rule of five, can increase the bioavailability of the drug [40]. 

Calixarenes and their water-soluble derivatives, in particular, show good biocompatibility and non-cytotoxicity, which are important prerequisites for practical application as DDSs. Macrocycles can induce a biological response through different methods of action. These compounds can insert themselves into a cell membrane to destroy its integrity, act as a prodrug, or release drugs linked into its central cavity in response to an external stimulus. Then, calixarene-binding pharmacologically active substituents become the vehicles that deliver the drug to a specific destination. Once the target is reached, local conditions, typically pH or enzymatic action, start the cleavage of the bond to release the active drug while the macrocycle is chemically degraded or excreted. These extrinsic stimuli break the non-covalent interactions between the drug and calixarene (π-π, CH-π, electrostatic interaction, H-bond and hydrophobic interaction), resulting in prolonged drug release and suggesting its potential application as a delivery system [30]. In the case of drug release into the tumor microenvironment, the mechanism is mainly pH dependent. Many studies have shown that the pH of cancer cells is between 5.7 and 7.8, while healthy tissue has a pH of 7.4. The difference in pH between normal cells and cancer cells is therefore useful for the controlled and localized administration of anticancer drugs [41]. The exceptional structural properties make the calixarene a viable candidate against cancer.

## 4. Application of Calixarene on Cancer Therapy

Cancer is one of the riskiest diseases for human health. The progression of cancer occurs through several steps. Initially, the cells of a certain tissue accumulate mutations in their genome and begin to acquire autonomous growth characteristics that lead to the formation of a primary tumor mass. The mass can remain localized and contained by a fibrous capsule or invade organs and tissues distant from the primary site, or at last, lead to metastasis. Non-surgical anticancer treatments (mainly conventional chemotherapy, targeted biological therapies and radiotherapy) have shown no complete efficacy because of their limits. Many of the drugs used show resistance phenomena, inadequacy to effectively deal with metastatic forms, and above all, low selectivity towards cancer cells. In particular, the poor selectivity of chemotherapeutic agents in use reduces their efficacy and causes severe systemic adverse reactions [42]. Another disadvantage of some anticancer drugs is poor water solubility and low stability, a condition that further limits their effectiveness. Thanks to its chemical–physical characteristics, calixarene can specifically target cancer cells and release the drugs slowly [43]. The best applications of calixarene in oncology derive from its ability to deliver chemotherapeutic agents with strong antitumor activity but whose use is limited due to their strong toxicity or poor water solubility. Although widely used anticancer drugs are used to counteract the proliferation of tumor cells, they are characterized by a low selectivity towards cancer cells. The action of anticancer agents against healthy cells leads to serious adverse effects thus limiting the effectiveness of anti-cancer therapy. The macrocyclic structure of calixarene allows it to host these drugs and release them in tumor tissues rather than in healthy tissues, thus decreasing the adverse effects related to low selectivity. The increased selectivity towards tumor cells allows the use of a smaller quantity of drug than would be required with the administration of free drugs. This is another factor that reduces toxicity [7].

The mechanism that allows calixarene macromolecules to selectively release the drug into cancer cells is threefold. In particular, the macrocomplex releases the anticancer drug only when the pH of the microenvironment is between 5.7 and 7.8, the pH typical of cancer cells. In addition, some in vivo studies have shown that the tumor environment is characterized by a high degree of hypoxia [44,45]; for this reason, using a supramolecular approach, some researchers have synthesized an azocalixarene sensitive to the hypoxic environment capable of hosting various anticancer drugs. The calixarene–anticancer drug complex dissociates in a hypoxic environment like that of the tumor, because in hypoxic conditions there is a reduction of azo-groups which leads to the dissociation between calixarene and the drug. The mechanism allows the controlled and localized release of the anticancer agent [43]. Another in vivo mechanism studied that increases the selectivity towards cancer cells and thus reduces the side effects of anticancer drugs is linked to the enhanced permeability and retention (EPR) effect. The EPR effect is based on the use of higher 40 kDa macromolecules capable of crossing vascular endotheliums with enhanced damaged permeability such as tumoral ones accumulating in the tumor mass. Thanks to the presence of junctions in healthy endotheliums, macromolecules containing tumor drugs are unable to cross them, thus allowing selectivity [46].

Numerous researchers have evaluated the efficacy of calixarene complexed with anticancer drugs such as intercalating agents, antimitotic agents and alkylating agents. Moreover, in addition to carrier usage, it has been shown that the derivatives of calixarene have antitumor activity [47]. For instance, they act as antagonists of the platelet-derived growth factor (PDGF) which exerts its biological activity by binding to the PDGFR, a receptor with tyrosine kinase activity (RTK). PDGFR is involved in angiogenesis and therefore in the formation of new blood vessels responsible for tumor progression [41]. Calixarenes have also been used in chemotherapy as calixarene-based gene delivery vectors to improve transfection efficiency and reduce cytotoxicity through functionalization with biocompatible substitutes. Calix [4] derived arenes used for in vitro gene transfection studies were obtained by adding guanidinium groups to the upper and lower edges. These were then co-assembled with diole-oylphosphatidylethanolamine (DOPE) to build the nanocarrier for plasmid delivery. These supramolecular nanosystems have demonstrated greater transfection efficiency than commercially available LTX lipofectamine agents in the treatment of human rhabdomyosarcoma. The role of calix [6] arene derivatives with six imidazole groups on the stabilization of the tetrameric structure of the tumor suppressor protein p53 was also investigated. The mutation and destabilization of the tetrameric structure of p53 is in fact related to many diseases and disorders, so its stabilization has been shown to improve the transcriptional activity of p53 through the formation of oligomers in the cells. These studies showed that calix [6] arene with adequate substitutions could represent a valid candidate for cancer therapy, restoring the function of mutant p53 [26].

### 4.1. Encapsulation of Intercalating Agents in Calixarene

An intercalating agent is a molecule capable of transversely inserting itself between two nitrogenous bases present in the DNA chains. This binding promotes the formation of non-functional gene products and inhibits the synthesis of nucleic acids inducing programmed death of cancer cell [48]. Among the most commonly used intercalating agents in chemotherapy is doxorubicin (DOX), an antibiotic isolated from *Streptomyces*, effective in the treatment of breast cancer, various forms of acute leukemia and malignant solid tumors. In addition to acting in the nucleus by intercalating DNA, DOX can be converted into a semichinone in the cytosol generating reactive oxygen species (ROS) by promoting oxidative stress. Although DOX has these therapeutic benefits, its use is limited by the risk of cardiotoxicity [49]. In particular, the toxic effects are dose-dependent, because DOX determines a reduction of endogenous antioxidants causing oxidative stress, especially when the cumulative dose exceeds 500 mg/m^2^. Oxidative damage to cardiac myocytes is associated with a reduction in the levels of antioxidant enzymes compared to other tissues [50]. DOX shows gastrointestinal toxicity and nausea; in addition, oxidative stress is also the cause of harmful effects on the nervous system [51]. To reduce the toxicity of DOX and simultaneously preserve its powerful antitumor activity, one strategy is to use nanostructures, such as calixarene, capable of encapsulating the drug and improving transport to the target. A previous in vitro study using A549 (adenocarcinomic human alveolar basal epithelial cell line), H358 (human lung cancer cell line), HepG2 (human liver cancer cell line) LS180 (adenocarcinomic human colonic epithelial cell line), MCF7 (breast cancer cell line) cell lines and on calf thymus DNA, shows that the encapsulation of DOX (positively charged at physiological pH) in molecules of *p*-sulfocalix [6] arene improved selectivity and reduced adverse effects of doxorubicin. In addition, spectroscopy studies revealed that calixarene alone is able to interact with the DNA structure. This is an additional advantage that improves the localization at the site of action and the approach of the DOX when it is encapsulated. The interaction is facilitated by the high flexibility of calixarene which can assume different conformations and interact with the polynucleotide (Table 1) [52].

**Figure 2:**
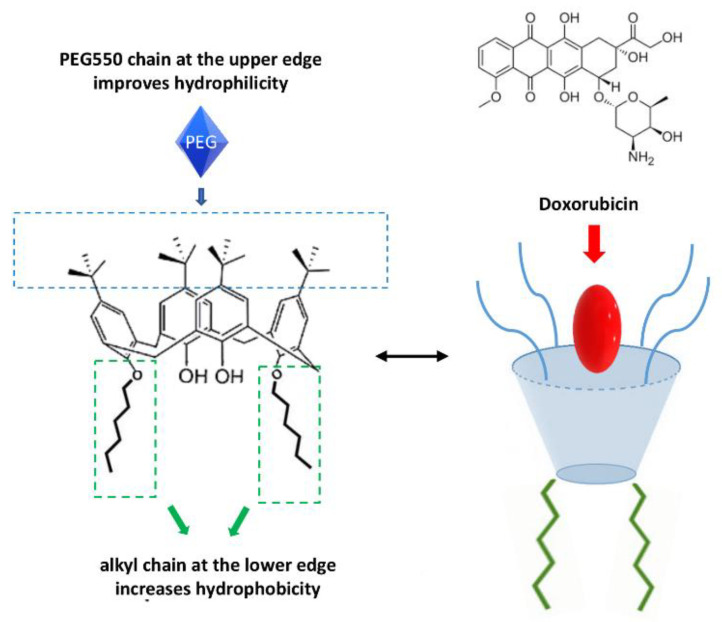
Schematic representation of DOX encapsulated in the calixarene structure.

By making structural changes to the calixarene molecule, it is possible to improve its biological and carrier activity. In particular, some researchers have introduced an alkyl chain that increases hydrophobicity and a PEG550 chain at the upper edge which improves hydrophilicity [58]. This amphiphilic structure allows the self-assembly of DOX, while the addition of folic acid improves the selectivity towards cancer cells because they are characterized by an overexpression of folate receptor (Figure 2). 

Performing a cell viability MTT assay [3-(4,5-Dimethylthiazol-2-yl)-2,5-Diphenyltetrazolium Bromide], the cytotoxicity of micelles on tumor cell lines (A549, HCT116, MDA-MB231) showed a higher antitumor activity compared to the free DOX formulation. To study the release kinetics of DOX from micelles in vitro, the method of dialysis in phosphate buffered saline solution, at various pH conditions, was performed. Initially the DOX fraction present between the hydrophilic and hydrophobic portion of the micelle is released rapidly, then, the DOX fraction encapsulated in the micelle core is released slowly for 24–48 h [59]. Another study conducted on doxorubicin-containing liposomes shows that this DDS (unlike calixarene) can encapsulate 89% of DOX and release 41% of it in a controlled manner for six hours. A comprehensive mathematical model of drug release kinetics for nano-liposomes has been derived from optimization studies of cationic PEGylated liposomal doxorubicin formulations for drug-gene delivery. Alternatively, nanovesicles of *p*-sulfonatocalix [4] arenes can encapsulate 86.0% of DOX, and the charged DOX molecules are released with increasing temperature together with the disassembly of the vesicles [60].

A recent cancer therapeutic strategy is to combine chemotherapy with therapeutic genes. The limit of this approach is correlated to a possible antagonism between therapeutic genes and drugs. A very recent in vivo study shows the development of a calixarene nanoparticle capable of carrying DOX and plasmid DNA targeting miR-21. The complex reduces the interference between drug and gene, improving stability and selectivity towards the therapeutic target. The observed anticancer effect is better in mice treated with the calixarene-DOX-DNA plasmid complex [61]. 

### 4.2. Encapsulation of Antimitotic Agents in Calixarene

Other anticancer drugs that can be delivered by calixarene are paclitaxel (PTX) and docetaxel (DTX), molecules belonging to the category of taxanes. They are used for the treatment of ovarian cancer, breast cancer, colorectal cancer and squamous cell carcinoma of the urinary bladder [62]. Taxanes are molecules of natural origin classified as antimitotic chemotherapeutic agents; they act by preventing the depolymerization of the mitotic spindle, causing the arrest of mitosis and the cell cycle of cancer cells [63]. Among the main adverse effects of PTX is the risk of hypersensitivity reactions, neurotoxicity and cardiotoxicity [64]. Another limitation of PTX is its poor solubility in water [65]. To overcome these problems, various studies demonstrate that efficacy and safety are improved when PTX is encapsulated in calixarene molecules [66,67]. For this purpose, a tetra-hexyloxy-*p*-sulfonate calix [4] arene (SC4–C6) amphiphilic derivate was synthesized using a thin-film sonication method. This formulation is capable of loading PTX with a gradual release at the site of action. A study with human cervical cancer cell cultures reveals a better cytotoxicity than free Taxol due to its greater absorption at the action site (Table 1) [53]. Moreover, PTX can be used in the treatment of human ovarian adenocarcinoma; studies show that its activity improves when PTX is encapsulated in a calix [4] arene conjugated *p*-phosphonate vesicle with folic acid (PCV) (Table 1). Furthermore, polyethylene glycol (PEG) molecules have been introduced into the structure as a spacer between folic acid and the surface of the vesicles in order to improve their water solubility and increase the drug’s half-life. Folic acid was added to increase target selectivity because human ovarian adenocarcinoma cells over-express folate receptors [54]. Several studies were performed to quantitatively compare the drug loading content of calixarene with other DDSs. It is important to highlight that drug loading content is closely related to the type of derivative and the type of interaction between calixarene and the host drug. For example, two types of amphoteric derivatives, calix [6] hexa-carboxylic acid (C6HCA) and calix [8] octocarboxylic acid (C8OCA) arene, show a good carrying capacity of paclitaxel (PTX), with a loading content of drug of 7.5% and 8.3%, respectively. HPLC analysis was performed to quantify the drug loading content of calixarene. In addition to considering the amount of encapsulated drug, it is important to consider the amount of drug released. The release profile of PTX-C6HCA is 33.7% within 30 min, while that of PTX-C8OCA is only 18.8% at 30 min [53]. Some studies on the amount of loading of PTX in other DDSs, other than calixarene, show that by associating PTX with the hyper-ramified hydrophilic poly (ether-ester) (HPEE), it can self-assemble into micelles with the capacity to contain from 4.1% to 10.7% of anticancer drug. Other DDSs are the solid lipid nanoparticles (SLNs). Two types of SLNs have been developed to host PTX: F68 (poloxamer F68)-SLN and Brij78 (polyoxyethylene 20 stearyl ether)-SLN with the ability to load 58.2% and 75.4% of PTX, respectively [68]. 

Another taxol-derived agent with antimitotic activity is docetaxel (DTX). It is active against various cancers such as prostate cancer, metastatic glioblastoma, metastatic breast cancer and lung cancer [69]. Frequent phenomena of resistance limit its use, linked above all to the overexpression of the drug’s efflux pumps. Another limitation is the low water solubility typical of these compounds [65]. However, the use of nano-carriers could be advantageous to reduce the adverse effects and overcome the problem of the low solubility of DTX. A macromolecular nanosystem is synthesized by combining the efficacy of two potent carriers: β-cyclodextrin heterodimers (βCD, with hydrophilic characteristics) and calix [4] arene (CA4, with hydrophobic characteristics). The complex can optimally host DTX and release it in a controlled way.

The cytotoxic effect of DTX encapsulated in the βCD-CA4 macro-complex has been studied on prostate and glioblastoma tumor cell lines, demonstrating that toxicity towards tumor cells is significantly greater than free DTX (Table 1) [55].

### 4.3. Encapsulation of Alkylating Agents in Calixarene

It has been reported that calixarene can encapsulate one more category of anticancer drugs, that being alkylating agents. This group of chemotherapy acts by inserting alkyl groups to the DNA chain, thus promoting the breaking of the strands and the inhibition of synthesis [70]. Among these drugs, the one most studied to be encapsulated in calixarene vesicles is carboplatin, a chemotherapeutic agent with a broad spectrum of activity in a number of malignancies, including gynecological cancers, germ cell cancers, head and neck cancers, chest cancers and bladder cancer [71]. In particular, carboplatin was easily associated with vesicles of tetra-para-phosphonomethyl calix [4] arenes containing *n*-hexyl fractions attached to phenolic oxygen centers. The release profile of carboplatin has been studied on ovarian cancer cells, demonstrating that the vesicles can optimally release the drug at pH 5.5, characteristic of cancer cells, thus improving the selectivity of the drug (Table 1) [72]. 

**Figure 3:**
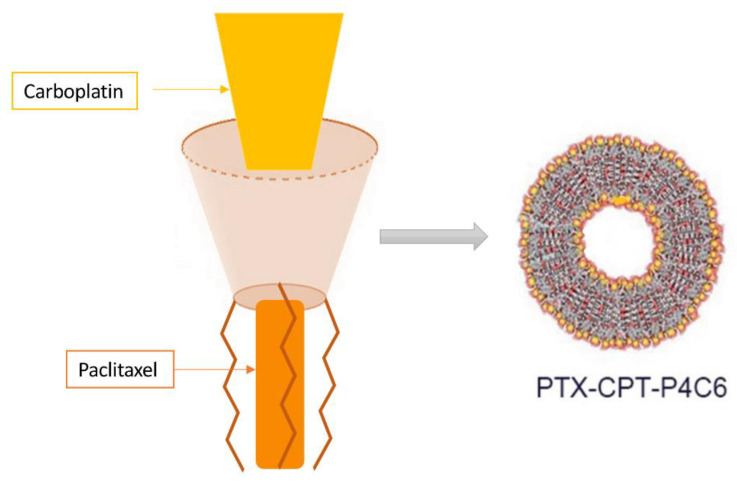
Schematic representation of the calyxarene structure capable of hosting both CPT and PTX.

Carboplatin is often used in anticancer therapy in combination with paclitaxel. The combination of these two anticancer drugs mediates a synergistic effect and reduces carboplatin-resistance, but at the same time increases toxicity [73]. For this purpose, nanoparticles of amphiphilic calixarene phosphonates capable of hosting both carboplatin and paclitaxel have been synthesized (Figure 3). 

This formulation mediates a synergistic effect and avoid side effects. The effects of this macro-complex have been studied on human colon cancer cell lines (HT-29 cells and Caco-2 cells) showing greater growth inhibition compared to free drugs [67]. The combination of carboplatin and paclitaxel was found to be effective for the treatment of ovarian cancer. For this reason, the two drugs have been encapsulated in new *p*-phosphonate conjugated folic acid-PEG calix [4] arene nanoparticles (Fp-PCN) and tested on both A549 cells and ovarian (SKOV3) cancer cells and in vivo. By using the ATP bioluminescence assay, it has been observed that the formulation inhibits the proliferation of tumor cells in a dose-dependent manner more than conventional drugs. Furthermore, a gene expression study was performed on SKOV-3 cells, showing that in the groups treated with FpPCNPAC + CAR there was a reduction in the expression of genes involved in tumor progression such as JMJD3 (Jumonji domain-containing protein D3), HER2 (Human epidermal growth factor receptor 2), RB1 (The retinoblastoma susceptibility gene), and BCL-2 (B-cell lymphoma 2) [72].

In particular, JMJD3 plays an oncogenic role in various tumors such as cancer of the kidneys, breast, prostate, skin, hematopoietic system, melanoma, Hodgkin’s lymphoma (HL), myelodysplastic syndrome (MDS), esophageal squamous cell carcinoma, and ovarian cancer [74].

Another application of calixarene is its association with temozolomide (TMZ), an alkylating drug agent for the treatment of glioblastoma. Although TMZ is stable at acidic pH, under slightly alkaline conditions it is rapidly hydrolyzed to MTIC (5-(3-methyltriazen-1-yl) imidazole-4-carboxamide) which, although still active, is unable to cross the blood brain barrier and is rapidly metabolized to an inactive derivative [75]. To achieve effective anticancer effects, high doses of TMZ must be administered resulting in severe side effects [57]. For these reasons, previous works have already demonstrated the possibility of stabilizing TMZ through its encapsulation in both nanoparticles and nanoliposomes [76,77]. Among the many possibilities, the encapsulation of TMZ in amphiphilic nanoparticles demonstrates greater stability compared to the free drug, based on chitosan and polylactic enriched with carboxy acids [78]. 

**Figure 4:**
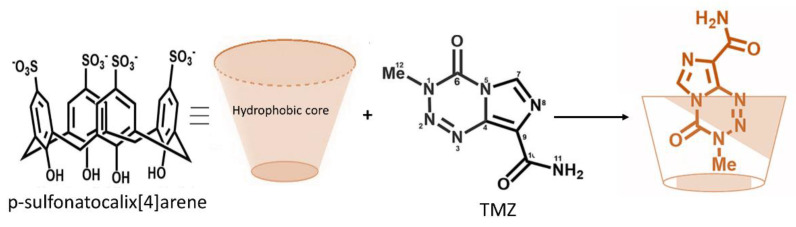
Schematic representation of TMZ encapsulated in the *p*-sulfonatocalix [4] arene macromolecule. The hydrophobic core of *p*-sulfonatocalix [4] arene interact and protect the methyl group of TMZ.

Subsequently, with the development of more advanced technologies, new formulations have been explored. One such innovative combination is a macromolecule where TMZ is encapsulated in *p*-sulfonatocalix [4] arene. In addition, this carrier has a hydrophobic center which protects the methyl group of the imidazotetrazine ring, thus preventing its rapid hydrolysis [68]. The protection of the methyl group is important because it is responsible for the alkylation of DNA, with consequent antitumor effects [79] (Figure 4). The activity of TMZ and TMZ associated with calixarene was studied in primary cultures of glioblastoma, and led to observations that cell growth was significantly reduced when treated with TMZ-calix compared to treatment with free TMZ (Table 1) [57]. 

## 5. Conclusions

In this review, we highlighted how calixarene can represent a valid carrier for anticancer drugs. Due to its high flexibility, calixarene can undergo numerous conformational changes and encapsulate various drugs used in traditional anticancer therapy, increasing their selectivity towards cancer cells and reducing their adverse effects. Although numerous drugs have been developed for the treatment of cancer in recent years, encapsulation in calixarene has only been investigated for conventional chemotherapeutics. For these reasons, future studies could explore the association of calixarene with anticancer drugs of new generations for target therapy.

## Figures and Tables

**Table 1:** This table summarizes the chemotherapy drugs designed to be encapsulated in calixarene.

Encapsulated Drug	Chemotherapy Class	Tumor Target	Reference
Doxorubicin	Intercalating agent	Adenocarcinomic human alveolar basal epithelial cell	
		Adenocarcinomic human colonic epithelial cell line	[52]
		Human lung cancer cell	
		Human liver cancer cell	
		Breast cancer cell line	
Paclitaxel	Antimitotic agent	Cervical cancer cells	[53]
		Human ovarian cells	[54]
Docetaxel	Antimitotic agent	Prostate tumor cell	[55]
		Glioblastoma tumor cell	
Carboplatin	Alkylating agents	Ovarian cancer cells	[56]
Temozolamide	Alkylating agents	Glioblastoma primary cells	[57]

## Data Availability

Not applicable.
